# Cyclic Dipeptide Shuttles as a Novel Skin Penetration Enhancement Approach: Preliminary Evaluation with Diclofenac

**DOI:** 10.1371/journal.pone.0160973

**Published:** 2016-08-22

**Authors:** Yousuf Mohammed, Meritxell Teixidó, Sarika Namjoshi, Ernest Giralt, Heather Benson

**Affiliations:** 1 School of Pharmacy, Curtin Health Innovation Research Institute, Curtin University, Perth, Western Australia, Australia; 2 Therapeutics Research Centre, The University of Queensland, School of Medicine, Translational Research Institute, Brisbane, Queensland, Australia; 3 Institute for Research in Biomedicine (IRB Barcelona), Barcelona Science and Technology Institute (BIST), Barcelona, Spain; Helsingin Yliopisto, FINLAND

## Abstract

This study demonstrates the effectiveness of a peptide shuttle in delivering diclofenac into and through human epidermis. Diclofenac was conjugated to a novel phenylalanyl-N-methyl-naphthalenylalanine-derived diketopiperazine (DKP) shuttle and to TAT (a classical cell penetrating peptide), and topically applied to human epidermis *in vitro*. DKP and TAT effectively permeated into and through human epidermis. When conjugated to diclofenac, both DKP and TAT enhanced delivery into and through human epidermis, though DKP was significantly more effective. Penetration of diclofenac through human epidermis (to receptor) was increased by conjugation to the peptide shuttle and cell penetrating peptide with enhancement of 6x by DKP-diclofenac and 3x by TAT-diclofenac. In addition, the amount of diclofenac retained within the epidermis was significantly increased by peptide conjugation. COX-2 inhibition activity of diclofenac was retained when conjugated to DKP. Our study suggests that the peptide shuttle approach may offer a new strategy for targeted delivery of small therapeutic and diagnostic molecules to the skin.

## Introduction

Considerable research effort has been focused on the development of skin penetration enhancement approaches primarily directed at overcoming the stratum corneum barrier. These approaches focus on improving the physicochemical characteristics of the permeant within the skin, facilitating transport by improved solubility or disrupting the skin barrier [1]. Several carrier-mediated delivery systems have recently been developed including nanoparticles and flexible liposomes. There is considerable debate around the efficacy and/or mechanism of enhancement of these technologies with current evidence suggesting that nanoparticles are most useful for targeting skin appendages as they do not effectively permeate through intact skin [2]. Flexible liposomes enhance permeation primarily due to their lipid, surfactant and ethanol components increasing solubility in skin, although some scientists suggest that they carry their payload through the skin barrier intact [3]. One approach that has received considerable attention is carrier-mediated drug delivery facilitated by cell-penetrating peptides [4–6], though the approach has received much less attention for enhancing skin permeation.

HIV-1-Trans-Activator of Transcription (TAT) was first reported to freely internalize into cultured cells in 1988 [7, 8] and this ability has been investigated and exploited for drug delivery. Since then many other cell penetrating peptides (CPP) have been described and evaluated for a range of applications [4–6]. CPPs are defined as peptides with a maximum of 30 amino acids, which are able to enter cells in a seemingly energy-independent manner [9]. The primary focus of research has been for delivery into the cytoplasm of cells throughout the body including targeting and crossing the blood-brain barrier and in targeting cancer cells. Hence these peptides have been termed “cell penetrating peptides” but also meet the broader definition of peptide shuttles. Rothbard et al [10] showed that conjugation of an arginine heptamer to cyclosporine A increased penetration into mouse and human skin, and provided a reduction in cutaneous inflammation. TAT mediated enhanced skin penetration has been reported for a number of compounds including lidocaine hydrochloride [11], salmon calcitonin [12], siRNA [13] and nanostructured lipid carriers [14]. However these studies were conducted in intact or disease compromised mouse or rat skin and are thus not comparable to human skin. Cohen-Avrahami et al. have investigated the interaction of a number of cell penetrating peptides with glycerol monooleate-based mesophases as potential dermal delivery systems [15–19]. They showed that TAT and penetratin enhanced delivery of diclofenac across porcine skin [17, 19].

Diketopiperazines (DKPs) are a class of biologically active compounds from a variety of natural sources including marine sponges, lichens and herbs that have exhibited antitumor, antiviral, antifungal, antibacterial, and antihyperglycaemic activities [20] and are generally considered safe [21]. We investigated these small cyclic peptides for targeted drug delivery [22, 23]. We synthesised a library of 15 DKPs with distinct side chains using a solid-phase synthetic methodology and evaluated their potential for enhanced delivery to the brain using the PAMPA *in vitro* model [22, 23]. Cyclic peptides occur naturally in the skin with roles in skin immunology, microbial defence, wound healing and pigmentation [24]. As cyclization of peptides has been shown to enhance skin permeation and/or stability [25, 26], we thought that DKPs will offer opportunities as peptide shuttles in skin delivery.

To explore this potential the effect of the specific DKP on the permeation of diclofenac has been assessed and is described in this manuscript. We chose the 2Nal cyclic peptide DKP Phe-*N*-MeNal based on our previous work that had shown this structure effectively permeated biological membranes [23]. Diclofenac is a non-steroidal anti-inflammatory drug (NSAID) that exerts anti-inflammatory, analgesic and anti-pyretic actions via inhibition of cyclooxygenase enzyme I (COX-1), and cyclooxygenase II (COX-2), with a four-fold higher selectivity for COX-2 [27]. It is also reported to inhibit the thromboxane-prostanoid receptor thus affecting arachidonic acid release and uptake; inhibit lipoxygenase enzymes and activate the nitric oxide-cyclic guanosine monophosphate pathway [28]. Diclofenac is indicated for a range of musculoskeletal conditions and in the treatment of actinic keratosis [29], pre-cancerous skin lesions induced by exposure to UV light. Goh and Lane [30] provide an excellent review of the topical administration of diclofenac and its salts.

We have explored the potential of DKP (Phe-*N*-MeNal) as a peptide shuttle enhancement strategy for skin delivery. Two approaches have been investigated: first that DKP will act as a carrier into and within the skin when covalently conjugated to diclofenac and, second that DKP will act within the skin to facilitate permeation of simultaneously applied diclofenac. In addition the anti-inflammatory activity of diclofenac on chemical conjugation with DKP was determined by measuring the COX-2 inhibition activity of diclofenac [31, 32] to demonstrate that the therapeutic activity is retained. TAT was included for comparison of the peptide shuttle with a classical cell penetrating peptide.

## Materials and Methods

### Chemicals and Instrumentation

Protected amino acids, handles and resins were supplied by Luxembourg Industries (Tel-Aviv, Israel), Neosystem (Strasbourg, France), Calbiochem-Novabiochem AG (Laüfelfingen, Switzerland), Bachem AG (Bubendorf, Switzerland), or Iris Biotech (Marktredwitz, Germany). PyBOP was supplied by Calbiochem-Novabiochem AG. DIEA, DIPCDI, DMAP, ninhydrin, and β-mercaptoethanol were obtained from Fluka Chemika (Buchs, Switzerland). PyAOP was supplied by Applied Biosystems and HOAt from GL Biochem Shanghai Ltd (Shanghai, China). Solvents for peptide synthesis and RP-HPLC were obtained from Scharlau or SDS (Barcelona, Spain). Trifluoroacetic acid was supplied by Kali-Chemie (Bad Wimpfen, Germany). Other chemicals used were purchased from Aldrich (Milwaukee, WI) and were of the highest purity commercially available. All commercial reagents and solvents were used as received, with the exception of DCM and DMF. DMF was bubbled with nitrogen to remove volatile contaminants and stored over activated 4 Å molecular sieves (Merck, Darmstad, Germany). DCM was passed through a short column of Al_2_O_3_ (in the case of DCM used for peptide synthesis). Diclofenac sodium (molecular weight 318.13 Da) was purchased from Sigma Aldrich (USA) and applied as the sodium salt in the epidermal diffusion experiments. TAT (molecular weight = 2020 g/mol) and TAT-diclofenac were synthesised by GLS Pharma (China) and characterised by HPLC and MS-MALDI. The diclofenac molecule was attached to the TAT peptide through an amide linkage at the *N*-terminus. Propylene glycol (PG), dimethyl sulphoxide (DMSO) and acetonitrile HPLC grade solvent were obtained from BDH Chemical Pty Ltd, Ajax FineChem and JT Baker respectively. Phosphate buffered saline solution (PBS) was prepared according to the United States Pharmacopoeia. COX enzyme assay kit was purchased from Cayman Ltd (USA).

### Synthesis and Purification of the DKP and DKP-Diclofenac Conjugate

**Fig 1:**
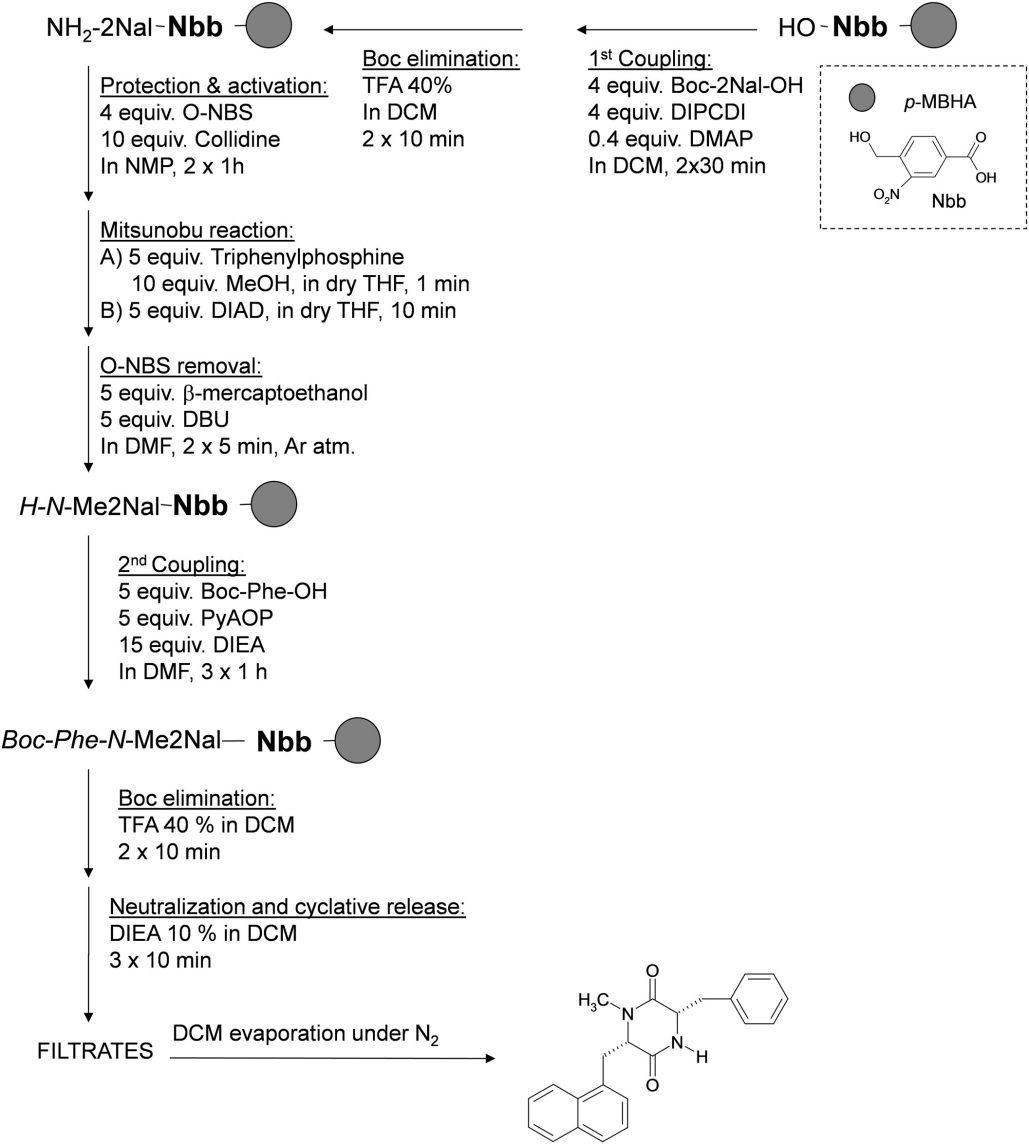
Synthesis of DKP Phe-*N*-MeNal.

**Fig 2:**
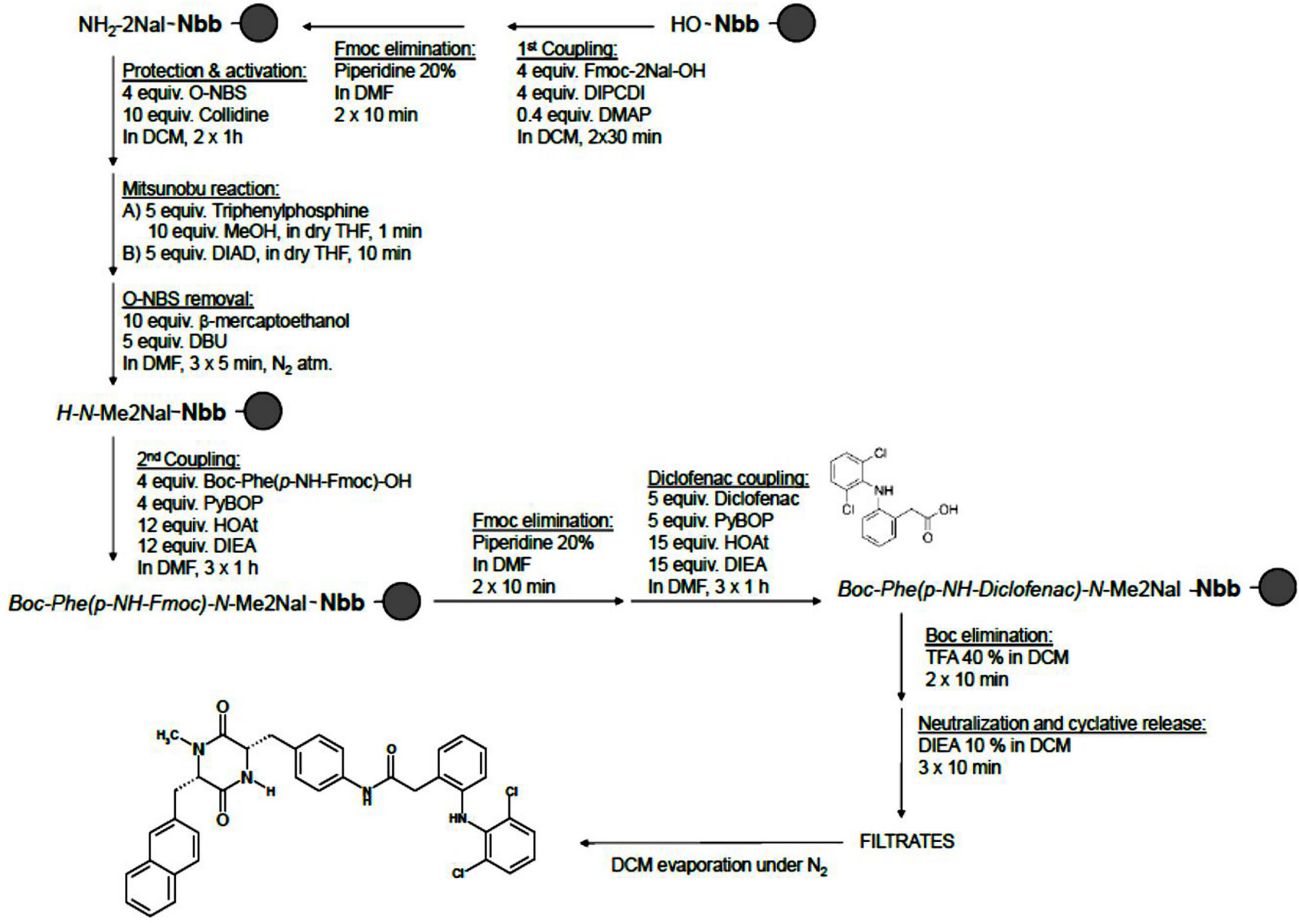
Synthesis of DKP Phe(Diclofenac)-*N*-MeNal conjugate.

Solid-phase methodology was used to synthesise the DKP Phe-*N*-MeNal (molecular weight = 359.44 g/mol), followed by DKP being covalently bound to diclofenac (molecular weight = 652.60 g/mol) (Figs 1 and 2)[23]. The purification was achieved by filtration and solvent evaporation under pressure (N_2_). The yield was 27% (95.8 mg) and 37% respectively. The molecular weight of all compounds synthesized was determined routinely using a MALDI-TOF Applied Biosystem 4700. 1 μL of peptide solution (0.1–1 mg/mL) mixed with 1 μL of α-cyano-4-hydroxycinnamic acid (ACH) matrix was seeded on the MALDI plate and air-dried. To prepare the matrix 10 mg of ACH was dissolved in 1 mL of MeCN/H_2_O 1:1 (v/v) containing 0.1% TFA.

HPLC chromatograms were obtained on a Waters Alliance 2695 with an automatic injector and a photodiode array detector 2998 Waters (Waters, Milford, MA) using a Sunfire C_18_ column (100 x 4.6 mm x 3.5 μm, 100 Å, Waters) and software EmpowerPro 2. The flow rate was 1 mL/min using MeCN (0.036% TFA) and H_2_O (0.045% TFA). 8-min linear gradients were used in all cases. HPLC chromatograms are provided in the Supplementary Material (S2 Fig).

All compounds were also analyzed using a high-resolution mass spectrometer to obtain their exact mass. Samples were dissolved in 200 μL of H_2_O:MeCN and diluted in H_2_O:MeCN 1% formic acid for MS analysis. The analysis was performed in a LTQ-FT Ultra (Thermo Scientific) and the sample was introduced by automated nanoelectrospray. A NanoMate (Advion BioSciences, Ithaca, NY, USA) infused the samples through the ESI Chip, which consists of 400 nozzles in a 20 x 20 array. Spray voltage was 1.7 kV and delivery pressure was 0.3 psi. MS conditions were: NanoESI, positive ionization, capillary temperature 200°C, tube lens 119V, ion spray voltage 2 kV and m/z 100–2000 amu.

#### Synthesis of Nbb Handle

4-Bromomethyl-3-nitrobenzoic acid (4.5 g, 17.3 mmols) was placed in a round-bottom flask provided with reflux system, and an aqueous NaHCO_3_ saturated solution was added (135 mL). The reaction was stirred and allowed to stand at 90–95°C. The progress of the reaction was monitored by TLC (chloroform/MeOH/AcOEt, 100:50:0.1, v/v) or by HPLC of an acidified aliquot in a gradient from 0 to 100% of MeCN in 15 min in a Symmetry C_18_ column (150 x 4.6 mm x 5 μm, 100 Å, Waters). Retention time for the final and initial products was 7.1 and 10.4 min respectively. The reaction was completed in 30 min. The solution obtained was hot-filtered, the reaction was quenched with HCl (12N) to pH 1–2, the crude product was extracted with AcOEt (3 x 100 mL), and the organic fractions were combined, washed with a saturated NaCl aqueous solution, and dried over anhydrous MgSO_4_. The AcOEt was evaporated in vacuo. The product was obtained as a pale yellow solid. Yield: 80%, 2.73 g. HPLC *t*_r_: 7.12 min (linear gradient 0–100% MeCN in 15 min). HPLC-MS: 196 Da. TLC: *Rf* = 0.3 (chloroform/MeOH/AcOEt, 100: 50:0.1, v/v). ^1^H NMR (CD_3_OD, 400 MHz, ᵟ ppm): 4.98 (s, 2H), 7.96 (d, *J* = 21, 1H), 8.26 (dd, *J* = 4, *J* = 20, 1H), 1.56–1.24 (m, 12H), 8.56 (d, *J* = 4, 1H).

#### General Protocols for Solid-Phase Peptide Synthesis

Syntheses were performed on a 100 μmol scale/each, in all cases L-amino acids were used. Solid-phase peptide elongation and other solid-phase manipulations were done manually in polypropylene syringes, each fitted with a polyethylene porous disk. Solvents and soluble reagents were removed by suction. Washings between synthetic steps were done with DMF (5 x 0.5 min) and DCM (5 x 0.5 min) using 5 mL of solvent/g of resin each time. During couplings the mixture was allowed to react with intermittent manual stirring.

**Identification Tests:** The Kaiser colorimetric assay was used for the detection of solid-phase-bound primary amines [33], while the De Clercq test was used for secondary amines bound to solid-phase [34].

**Initial Conditioning of Resin:** The *p*-MBHA resin was conditioned by washing with DCM (5 x 30 s) followed by a 40% TFA solution in DCM (1 x 30 s and 2 x 5 min). This acid treatment was followed by a neutralization step with DIEA 5% in DCM (3 x 2 min) and finally the resin was washed with DCM (5 x 30 s).

**Coupling of Nbb Handle to p-MBHA Resin:** Nbb handle (97 mg, 5 equiv) in DCM (3 mL/g of resin) and DIPCDI (77 μL, 5 equiv) were sequentially added to the *p*-MBHA resin (164 mg). The mixture was left to stir overnight. The solvent was then removed by suction, the resin was thoroughly washed, and the reaction was monitored with the Kaiser test.

**Boc Group Removal:** The Boc group was removed by treating the resin with 40% (v/v) TFA in DCM (3–4 mL/g of resin, 2 x 10 min).

**Fmoc Group Removal:** The Fmoc group was removed by treating the resin with 20% piperidine in DMF (3–4 mL/g of resin, 2 x 1 min and 1 x 20 min).

#### Coupling Methods

**Method 1, Coupling of the First Amino Acid onto the Nbb Handle:** Amino acid derivative (4 equiv), DIPCDI (62 μL, 4 equiv), and DMAP (5 mg, 0.4 equiv) in DCM (1–3 mL/g of resin) were sequentially added to the resin. The mixture was allowed to react with intermittent manual stirring for 30 min. The solvent was then removed by suction, the resin was thoroughly washed, and the coupling was repeated once.

**Method 2, Coupling of the Second Amino Acid:** Protected amino acid (5 equiv) and PyAOP (260 mg, 5 equiv) in DMF (1–3 mL/g of resin) were sequentially added to the resin followed with DIEA (255 μL, 15 equiv). Alternatively, protected amino acid (4 equiv), PyBOP (4 equiv) and HOAt (12 equiv) in DMF (1–3 mL/g of resin) were sequentially added to the resin followed with DIEA (12 equiv). The mixture was left to react with intermittent manual stirring for 1 h. The solvent was removed by filtration, the resin was washed as indicated above, and the coupling was repeated two more times. The extent of the coupling was checked by the De Clercq test.

#### Amino Acid N-Alkylation

The *N*-methylation of the amino acid derivatives was performed using the methods described by Biron et al. [35] and Yang and Chiu [36] This process can be divided in to three steps: (A) protection and activation with O-NBS, (B) Mitsunobu reaction, and (C) O-NBS removal:

1. **Protection and Activation with O-NBS:** To perform the protection, O-NBS (88 mg, 4 equiv) and collidine (170 μL, 10 equiv) in NMP were added to the resin. The reaction was left with intermittent manual stirring for 1 h and this step was repeated once and checked by the Kaiser test.
2. **Mitsunobu Reaction:** The Mitsunobu reagents, triphenylphosphine (133 mg, 5 equiv), and MeOH (24 μL, 10 equiv) in dry THF were added to the resin and left for 1 min; afterward, without filtering, DIAD (98 μL, 5 equiv) was added in dry THF and left for another 10 min.
3. **O-NBS Removal:** To proceed to O-NBS removal, β-mercaptoethanol (70 μL, 10 equiv) and DBU (75 μL, 5 equiv) in NMP were added to the resin, and the mixture was left to react for 5 min under an argon atmosphere. This process was repeated once.

#### Coupling of Diclofenac

The corresponding DKP provided with a NH_2_ group was used, and was reacted with diclofenac-COOH (5 equiv), using PyBOP (5 equiv) and HOAt (15 equiv) as coupling reagents and DIEA (15 equiv) as a base in DMF for 1 h. This process was repeated twice.

#### Neutralization-Cyclization and Cleavage All in One Step

Only by treating the resin with 10% DIEA in DCM (3 x 10 min) was neutralization-cyclization and cleavage accomplished. The filtrates were collected, DCM was evaporated under N_2_, and the residue was solved in H_2_O:MeCN (1:1) and lyophilized.

#### DKP and DKP-Diclofenac Purification

DKP and DKP-diclofenac were purified in a Waters system with MassLynx 4.1 software, a 2545 binary gradient module, a 2767 manager collector, a 2998 photodiode array detector and a Sunfire C_18_ column (150 x 10 mm x 3.5 μm, 100 Å, Waters). The flow rate was 6.6 mL/min using MeCN (0.1% TFA) and H_2_O (0.1% TFA). Purity was checked by analytical reverse-phase HPLC. MALDI-TOF MS and LC-MS were used to confirm the identity of the compounds synthesized, and purity was assessed by HPLC-UV at 220 nm using our previously described methodology [37]. Briefly, peptides containing N-methyl amino acids are known to show a complex profile. In our case, the peaks were inter-convertible. When the main peak was collected, lyophilized and reinjected, two peaks appeared. The compound was considered as a whole (i.e. composed of the two peaks) [37].

### Diffusion Studies

#### HPLC Instrumentation and Conditions for Skin Diffusion Samples

The samples obtained after *in vitro* skin permeation studies were analysed by reverse phase HPLC (Agilent 1200 system with a binary pump (G1312A), autosampler (G1329A), degasser (G1379B) and photo diode array detector using optimum wavelengths (G1365B). Separation was achieved on a Jupiter C_18_, 300 Å column (5 μm, 4.6 mm × 150 mm; Phenomenex) using gradient elution of solvents (diclofenac, DKP: mobile phase A 0.045% TFA in water and B 0.036% TFA in acetonitrile linear gradient 10–100% phase B over 10 min; TAT: mobile phase A 0.1% TFA in water and B 0.1% TFA in acetonitrile linear gradient 10–100% phase B over 22 min). Integration was undertaken using Chemstation software. Gradient elution at 1 mL/min was used for all compounds. HPLC methods were validated for linearity, precision, intra and inter day repeatability and accuracy. Lower limits of detection and quantification were determined for all the actives.

#### Human Skin Preparation

Full thickness human skin samples from patients (26–48 years old females) undergoing abdominoplasty at Perth (WA, Australia) hospitals were refrigerated immediately after surgery. Sampling was approved by the Human Research Ethics Committee of Curtin University and was conducted in compliance with the guidelines of the National Health and Medical Research Council of Australia. Epidermal sheets were obtained and stored as previously described [38].

#### *In Vitro* Skin Diffusion Studies

*In vitro* permeation studies across human epidermis were performed in Pyrex glass Franz-type diffusion cells (skin cross sectional area 1.18 cm^2^; receptor volume approximately 3.5 mL) using our established methods [39–41] and a receptor solution of 25:75 PG:PBS. Epidermal membrane integrity was determined by a digital multimeter, with membranes exhibiting an electrical resistance of less than 20 kΩ rejected from the study [39–41]. The donor solutions (300 μL) containing 150 μg DKP or TAT, 1:1 physical admixture of DKP and diclofenac (150 μg each), or DKP or TAT conjugated to diclofenac (equivalent to 150 μg diclofenac), in 25:75 PG:PBS vehicle were added to the donor compartment, which was occluded to reduce evaporation. Two hundred μL samples of the receptor phase were withdrawn and replaced with an equal volume of fresh pre-warmed (37°C) 25:75 PG:PBS over an 8 h period and the content analysed by HPLC.

At least 5 replicates were conducted for each of the DKP, TAT, diclofenac, physical admixtures and diclofenac conjugates. The cumulative amount of CPP/shuttle or active permeated through the epidermis (μg/cm^2^) versus time (h) was plotted. The flux through the human epidermis was determined from the slope of the plot of cumulative amount versus time and expressed as μg/cm^2^/h. Permeability coefficients (cm/h) were calculated.

#### Mass Balance and Recovery

The uptake of the each TAT, DKP, diclofenac and the conjugates in the epidermis was determined by solvent extraction and HPLC analysis using a validated extraction procedure. Briefly, the skin sections were placed in 500 μL methanol and vortexed for 2 min, then transferred to a second 500 μL methanol volume and vortexed for another 2 min. The skin sample was then placed in 1 mL propylene glycol for 1 h with agitation. After centrifuging each extract at 10,000 × g for 10 min, the resultant supernatants were diluted appropriately, quantified by HPLC, and the data combined to give total penetrant in the epidermal section. The total amount extracted from the skin by the 3-step process was included in Table 1.

**Table 1:** Summary of permeation data for diclofenac and peptide shuttles.

Skin distribution					
Compounds	Applied dose (μg)	Amount extracted (μg)	Maximum amount permeated (μg/cm^2^)	Amount extracted from the skin (% of applied dose)	Amount permeated (% of applied dose)
DKP	150 μg	10.43(±2.04)	6.50(±1.94)	6.95	4.33
TAT		13.20(±2.21)	2.43(±1.00)	8.80	1.62
Diclofenac		8.58(±1.50)	0.91(±0.15)	5.72	0.60
DKP-diclofenac conjugate		138.07(±16.50)	6.01(±0.16)	92.04	4.00
TAT-diclofenac conjugate		20.25(±2.39)	3.00(±0.55)	13.5	2.00

Mass balance/recovery after the skin permeation experiments was determined by removing and retaining the donor solution, and washing the epidermal surface, donor and receptor compartments and retaining all washing solutions. The content of all solutions was analysed by HPLC following suitable dilution.

#### Cyclooxygenase (COX) Inhibition Assays

The activity of diclofenac was determined by assessment of COX-2 inhibition. DKP-diclofenac conjugate and diclofenac activity was assessed using a COX Fluorescent Inhibitor Screening Assay Kit (700100, Cayman Chemical, USA). The activity of diclofenac and DKP-diclofenac conjugate was assessed over a concentration range (0.000155–0.31 mM) with a 2 min incubation period and a fluorescence microplate reader equipped with standard fluorescein.

### Statistical Analysis

Skin permeation data consisted of cumulative measurements of the drug amount taken at various times (1 to 8 h) following active and passive treatments. A random effects regression model was used to compare the treatments at various time points, with the timings treated as categorical variables (so that no linear relationship was assumed between permeation and time). This type of regression model is equivalent to a repeated measures analysis of variance, in that it properly takes into account correlations between measurements made on the same sample at different time points. If the standard deviations of the permeations appeared to differ widely for different time periods, a logarithmic transformation was performed on the permeation before analysis.

Pairwise comparisons were calculated from the regression model by requesting certain ‘contrasts’ as required. Contrasts were tailored to make specific comparisons, and obtain the p-values for them. The standard errors on which the contrasts were based were estimated from the regression model that was based on all the available data. If the analysis was performed on the log-transformed data, the p-values for the pairwise comparisons were based on this analysis, but the mean permeation was quoted on the original scale (to simplify interpretation).

For the analysis of CPP/shuttle and CPP/shuttle conjugates retained in the skin, there were no repeated measurements, so a straightforward analysis of variance (ANOVA) was performed to compare results between treatments. A student’s t-test was performed for comparison of COX-2 inhibition by CPP/shuttle conjugates and diclofenac.

All statistical analyses were performed using SAS version 9.2 statistical software (SAS Institute Inc, Cary, NC, USA). A p-value <0.05 was taken to indicate statistical significance.

## Results and Discussion

We show that the novel peptide shuttle DKP increases permeation of diclofenac into and across human skin. DKP enhanced diclofenac permeation when conjugated but not as a physical admixture. We also show that when conjugated to the DKP, diclofenac retains its COX-2 inhibition activity. DKP was more effective than TAT in enhancing skin penetration of diclofenac.

### Synthesis and characterization of DKP and conjugate

DKP Phe-*N*-MeNal and DKP Phe(Diclofenac)-*N*-MeNal were synthesised and purified as described, providing a yield of 27% and 37% respectively. Characterization information and chromatograms are provided in S1 and S2 Figs).

#### Epidermal Penetration of TAT and DKP

**Fig 3:**
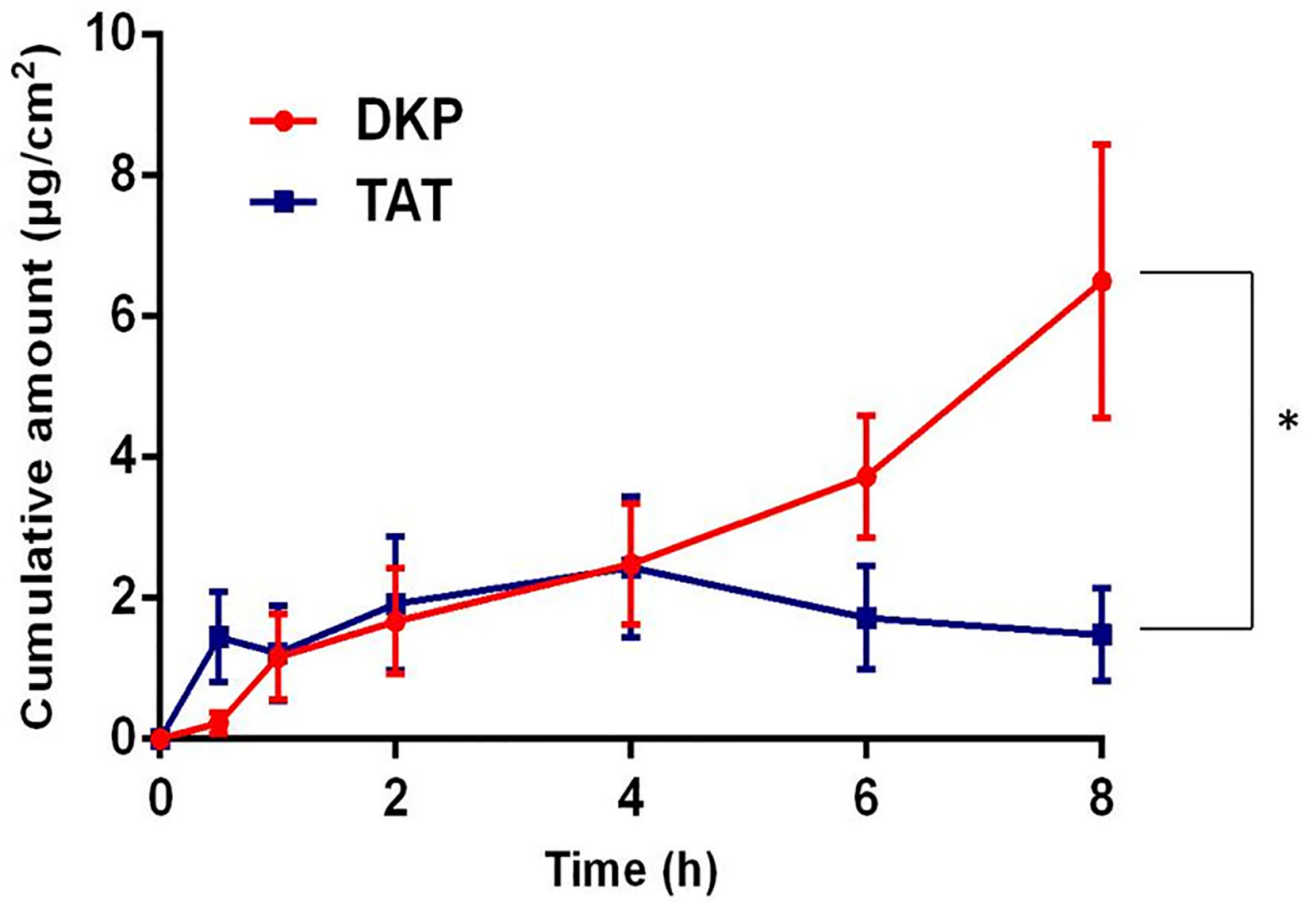
Comparison of human skin permeation profiles of DKP and TAT alone. Indicated values are mean ± (sem) of n = 7, *P<0.05.

We clearly demonstrate that TAT and DKP effectively permeate human epidermis following topical application (150 μg in 300 μL 25:75 PG:PBS) for 8 h (Fig 3).

The epidermal permeation of DKP was significantly higher than that of TAT (Table 1: p<0.05). Cumulative amount verses time, permeability coefficient and epidermal flux data are presented in Table 1.

Our finding is in agreement with previous studies that showed CPPs including TAT, cross the stratum corneum barrier [42]. Lopes et al. [43] reported rapid penetration of TAT (1559 g/mol) and a molecular modification of TAT (YARAAARQARA) named YARA (MW 1668 g/mol) in porcine ear skin *in vitro*. They also demonstrated that combination with the chemical penetration enhancer’s monoolein and oleic acid did not further enhance skin permeation of these CPPs. These studies were undertaken on whole skin that is not the accepted model membrane for human skin permeation studies [44]. In our study we demonstrate that DKP, from the novel diketopiperazine class of peptide shuttles, permeates into and through human epidermis. Whilst DKP has previously been shown to cross the blood-brain barrier [23], this is the first demonstration of its penetration across skin. DKP permeation into and through the skin was greater than TAT. DKP (MW 275 g/mol) is a much smaller molecule than TAT (1559 g/mol), which is likely to be an important factor in facilitating permeation through the highly ordered stratum corneum lipid structure. This is also likely to offer a significant advantage when coupled with a cargo that would further increase the overall size of the combined molecule.

In addition we have established the stability of DKP in human skin (unpublished data). This was expected as DKP is a cyclic peptide, a conformation which has been demonstrated to confer enhanced peptide stability in biological systems [25, 45–47] and in some cases can also enhance activity [48].

#### Influence of TAT on Epidermal Penetration of Diclofenac

**Fig 4:**
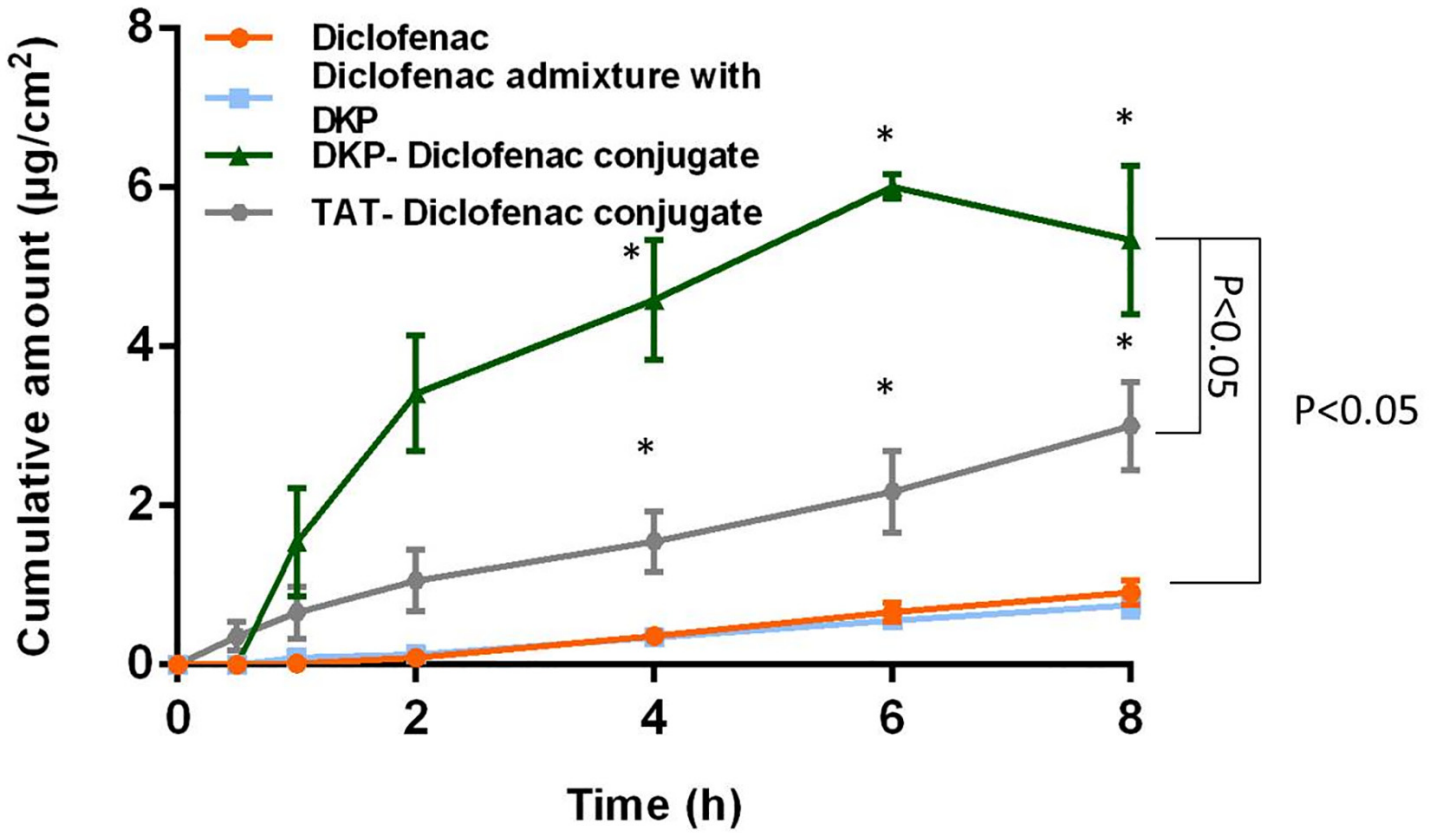
Human skin permeation of diclofenac alone, diclofenac as admixture with DKP, diclofenac as DKP conjugate and diclofenac as TAT conjugate. The data is represented as mean ± sem (n = 5), *P<0.05.

TAT was successfully conjugated to diclofenac and applied to human epidermis (300 μL containing 150 μg diclofenac, alone or as conjugate, in 25:75 PG:PBS vehicle) for 8 h. TAT-diclofenac conjugate enhanced the permeation in comparison to diclofenac alone (steady state flux 0.35 and 0.12 μg/cm^2^/h respectively: Fig 4; Table 1, p<0.05).

Lopes et al [43] reported rapid penetration of YARA and TAT in porcine ear skin *in vitro*. However when TAT was FITC labeled to aid quantification, the transdermal delivery of TAT-FITC reduced from 2.0 nmol/cm^2^ to 0.027 ± 0.009 nmol/cm^2^ at 8 h. This suggests that the increase in the molecular weight caused by the FITC reduced permeation of TAT. When TAT and YARA were conjugated to peptide P20 (Heat shock protein 20: WLRRASAPLPGLK; MW 2005 g/mol), skin penetration was slow and very limited despite the high permeation of the cell penetrating peptides alone.

In our study TAT increased the rate and extent of human epidermal penetration by 2-fold, however again there is a significant difference in molecular weight between the conjugated molecules in this study and the work of Lopes et al [43]. We would suggest that whilst TAT alone penetrates skin well and is capable of carrying a small molecule such as diclofenac across the skin, the work of Lopes suggests there is a molecular weight cutoff beyond which it is not an effective skin penetration enhancer.

#### Influence of DKP on Epidermal Penetration of Diclofenac

In this study we have demonstrated shuttle-mediated delivery of diclofenac when conjugated to DKP. DKP was successfully conjugated to diclofenac (via the reaction Fig 2) and applied to human epidermis (300 μL containing 150 μg diclofenac, alone or as conjugate in 25:75 PG:PBS vehicle) for 8 h. When conjugated to DKP, the permeation of diclofenac increased approximately 6-fold compared to diclofenac alone (steady state flux 0.75 and 0.12 μg/cm^2^/h respectively; p<0.05; Table 1 and Fig 4). DKP was a significantly more effective skin permeation shuttle of diclofenac than TAT (0.75 and 0.35 μg/cm^2^/h respectively; p<0.05).

In order to determine the mechanism by which DKP enhanced the epidermal penetration of diclofenac, a 1:1 admixture of DKP with diclofenac was compared to the conjugated DKP-diclofenac (Fig 4 and Table 1). The aim of this study was determine if the DKP facilitated permeation in co-administration or if DKP acted as a shuttle only when conjugated to the cargo molecule. When applied as an admixture there was no significant enhancement in the skin penetration of diclofenac. This suggests that DKP does not facilitate skin permeation by acting on the stratum corneum barrier per se, but rather acts as a carrier through the stratum corneum only when covalently conjugated to its cargo. This offers potential advantages for specific and targeted delivery and reduces the likelihood of the generalized skin irritancy caused by many chemical penetration enhancers.

#### Retention of TAT, DKP and Conjugates in the Skin

**Fig 5:**
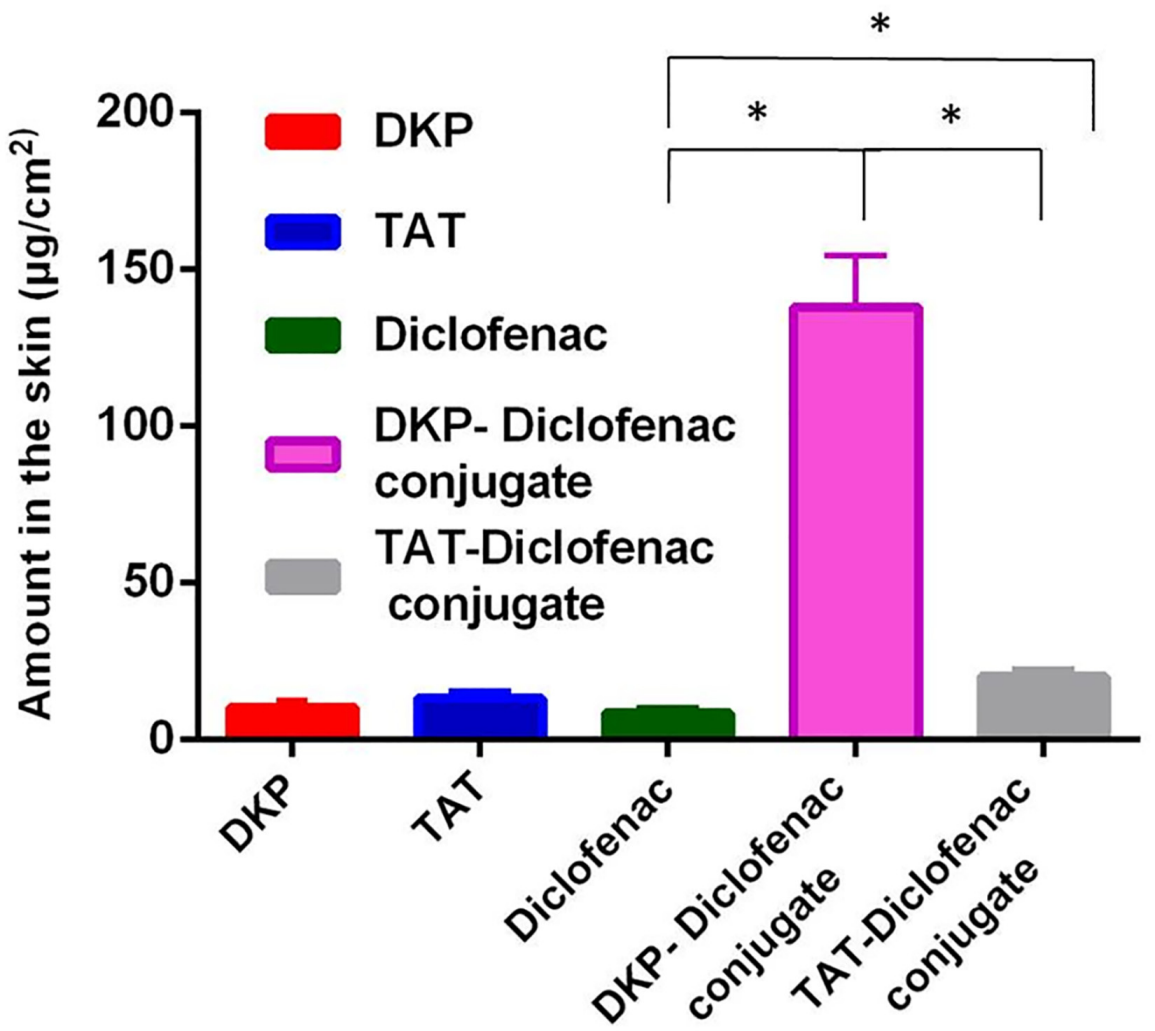
Amount of DKP, diclofenac, DKP-diclofenac conjugate, TAT-diclofenac conjugate and TAT in the skin after permeation experiments (donor amount of 150 μg). Data is represented as mean ± sem (n = 4), * P<0.05.

Diclofenac delivered across the epidermis would be expected to be available to exert a therapeutic effect. In addition, diclofenac remaining in the epidermis is expected to permeate over time to the deeper tissues; therefore contributing to the therapeutic effect. The diclofenac remaining in the epidermis following application of all compounds was determined. The amount of DKP-diclofenac conjugate accumulated in the epidermis was significantly higher than TAT-diclofenac conjugate, which was significantly greater than diclofenac alone (p<0.05; Fig 5). The extraction of all solutes from the epidermis was successfully achieved by two successive extractions with methanol, followed by propylene glycol, giving recoveries greater than 90%.

Clearly both TAT and DKP increased permeation of diclofenac into and through the epidermis. When TAT and DKP were applied without diclofenac, TAT accumulation was greater than DKP, though the difference was not statistically significant. Lopes previously reported TAT accumulation in porcine skin [43]. The DKP-diclofenac and TAT-diclofenac conjugate permeated into the skin to a significantly higher extent as compared to diclofenac alone (p<0.05). The amount of DKP-diclofenac in the skin was 7 times that of TAT-diclofenac conjugate (p<0.05).

In a recent clinical comparison of the effectiveness of topical application of methyl aminolevulinic acid with photodynamic therapy verses diclofenac in hyaluronic acid, it was found that although the diclofenac therapy is more cost effective, it is less clinically effective in the complete remission rates for treatment of actinic keratosis [49]. The ability of DKP to enhance diclofenac retention within the epidermis may be useful as an alternative diclofenac delivery method for targeting actinic keratosis lesions.

#### Effect of Conjugation to DKP on Inhibition of Cyclooxygenase (COX) by Diclofenac

**Fig 6:**
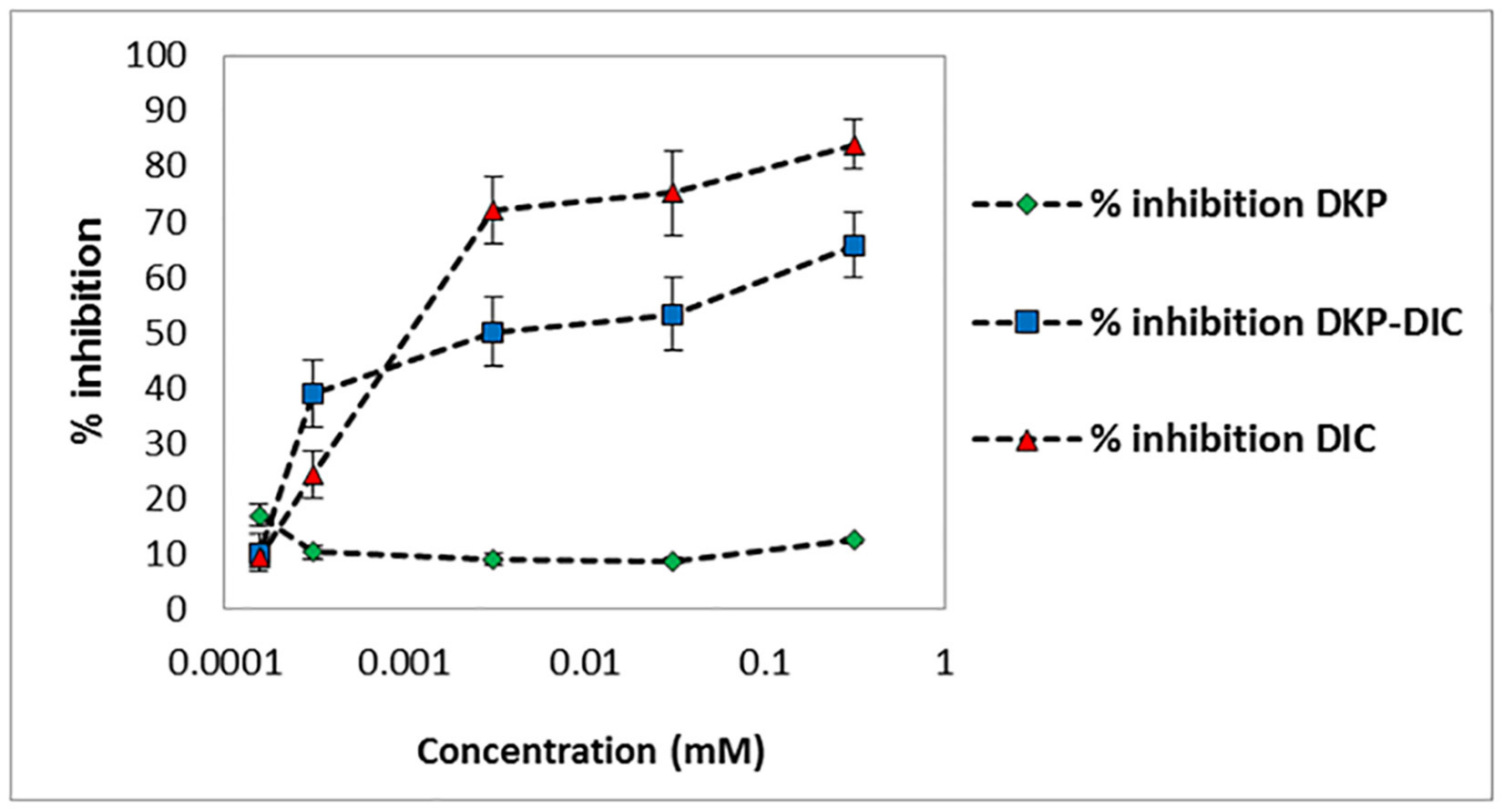
Percent inhibition of COX-2 by diclofenac,DKP-diclofenac conjugate and DKP alone over the concentration range of 0.00015–0.31 mM. The indicated values are means ± SD (n = 3). The COX-2 inhibition caused by diclofenac was tested for significance against DKP alone and DKP-diclofenac conjugate (* p<0.05).

COX-2 inhibition was substantially retained when diclofenac was conjugated to DKP (Fig 6). Over the concentration range of 0.000155–0.31 mM, the inhibition of COX-2 by DKP-diclofenac was reduced by approximately 20–30% compared to diclofenac alone (p<0.05). DKP itself did not demonstrate COX-2 inhibition. Statistically significant reduction in inhibition of COX-2 was also achieved by diclofenac alone when compared to DKP alone (p<0.05) whereas a significant difference in COX-2 inhibition was not found between DKP-diclofenac conjugate and diclofenac alone.

This provides a clear indication that the biological and thus therapeutic activity of diclofenac is not adversely affected by conjugation to DKP. Given the 6-7x increase in permeation into and through the epidermis it is expected that DKP mediated delivery would substantially enhance the therapeutic activity of diclofenac. Orally administered NSAIDs can cause complications, including ulcers and gastrointestinal bleeding therefore improved topical delivery of the NSAIDs that can target their site of action locally offers significant clinical advantages. Such localization would be extremely beneficial for the topical use of diclofenac in the treatment of actinic keratosis [50].

The DKP skin shuttle could also be used to conjugate many drugs including corticosteroids, antifungal and antibacterial agents. This would be advantageous for the treatment of skin conditions such as psoriasis, atopic dermatitis and for targeting drugs for the treatment of skin cancers where the adverse side-effects of the drugs when delivered systemically can cause considerable problems.

## Conclusions

DKP and TAT effectively permeated into and through human skin. When conjugated to diclofenac, both DKP and TAT enhanced delivery into and through skin, though DKP was more effective. Penetration of diclofenac through human epidermis was increased by conjugation to the peptide shuttle and cell penetrating peptide with enhancement of 6x by DKP-diclofenac and 3x by TAT-diclofenac. In addition the amount of diclofenac retained within the epidermis was significantly increased by peptide conjugation. COX-2 inhibition activity of diclofenac was retained when conjugated to DKP. Our study suggests that DKP may offer a novel strategy for targeted delivery of small therapeutic and diagnostic molecules to the skin.

## Supporting Information

S1 Fig DKP Phe-*N*-Me2Nal characterization. (PDF)

S2 Fig DKP Phe(Diclofenac)-*N*-Me2Nal conjugate characterization. (PDF)
