# Supplementary material for: Cyclic Dipeptide Shuttles as a Novel Skin Penetration Enhancement Approach: Preliminary Evaluation with Diclofenac
Source: PLoS One. 2016 Aug 22;11(8):e0160973. doi: 10.1371/journal.pone.0160973 (PMC4993479; doi:10.1371/journal.pone.0160973)
Supplement: S2 Fig — (PDF) [file pone.0160973.s002.pdf]

S2 Fig. DKP Phe(Diclofenac)-*N*-Me2Nal conjugate characterization

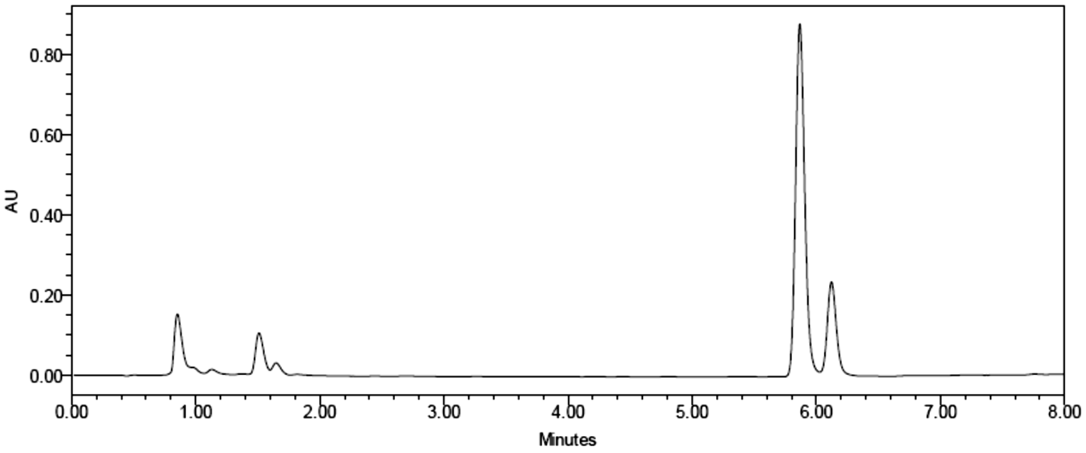

HPLC analysis: t_r: 5.8 min Gradient from 50-100% MeCN (with 0.036%TFA) in H_2O (with 0.045%TFA) in 8 minutes using a Sunfire-C_{18} (4.6 x 100 mm & 3.5 mm; flow: 1 mL/min); Purity: 95%; MALDI-TOF mass analysis: $[M+H]^+$ calculated 652.60 Da; $[M+Na]^+$ experimental: 673.18 Da, $[M+K]^+$ experimental: 689.13 Da; HR-MS: Theoretical 651.19242; Exp. 651.19333; Yield (Synthesis and Purification): 37%.
